# Risk of Cancer in Patients with Iron Deficiency Anemia: A Nationwide Population-Based Study

**DOI:** 10.1371/journal.pone.0119647

**Published:** 2015-03-17

**Authors:** Ning Hung, Cheng-Che Shen, Yu-Wen Hu, Li-Yu Hu, Chiu-Mei Yeh, Chung-Jen Teng, Ai-Seon Kuan, San-Chi Chen, Tzeng-Ji Chen, Chia-Jen Liu

**Affiliations:** 1 Department of Psychiatry, Kaohsiung Veterans General Hospital, Kaohsiung, Taiwan; 2 School of Medicine, National Yang-Ming University, Taipei, Taiwan; 3 Department of Psychiatry, Chiayi Branch, Taichung Veterans General Hospital, Chiayi, Taiwan; 4 Department of information magagement, National Chung-Cheng University, Chiayi, Taiwan; 5 Cancer Center, Taipei Veterans General Hospital, Taipei, Taiwan; 6 Institute of Public Health, National Yang-Ming University, Taipei, Taiwan; 7 Department of Family Medicine, Taipei Veterans General Hospital, Taipei, Taiwan; 8 Division of Oncology and Hematology, Department of Medicine, Far Eastern Memorial Hospital, New Taipei City, Taiwan; 9 Division of Hematology and Oncology, Department of Medicine, Taipei Veterans General Hospital, Taipei, Taiwan; University of Florida, UNITED STATES

## Abstract

**Objective:**

This study evaluated the risk of cancer among patients with iron deficiency anemia (IDA) by using a nationwide population-based data set.

**Method:**

Patients newly diagnosed with IDA and without antecedent cancer between 2000 and 2010 were recruited from the Taiwan National Health Insurance Research Database. The standardized incidence ratios (SIRs) of cancer types among patients with IDA were calculated.

**Results:**

Patients with IDA exhibited an increased overall cancer risk (SIR: 2.15). Subgroup analysis showed that patients of both sexes and in all age groups had an increased SIR. After we excluded patients diagnosed with cancer within the first and first 5 years of IDA diagnosis, the SIRs remained significantly elevated at 1.43 and 1.30, respectively. In addition, the risks of pancreatic (SIR: 2.31), kidney (SIR: 2.23), liver (SIR: 1.94), and bladder cancers (SIR: 1.74) remained significantly increased after exclusion of patients diagnosed with cancer within 5 years after IDA diagnosis.

**Conclusion:**

The overall cancer risk was significantly elevated among patients with IDA. After we excluded patients diagnosed with IDA and cancer within 1 and 5 years, the SIRs remained significantly elevated compared with those of the general population. The increased risk of cancer was not confined to gastrointestinal cancer when the SIRs of pancreatic, kidney, liver, and bladder cancers significantly increased after exclusion of patients diagnosed with IDA and cancer within the first 5 years. This finding may be caused by immune activities altered by IDA. Further study is necessary to determine the association between IDA and cancer risk.

## Introduction

Anemia is the most common micronutrient deficiency, affecting 24.8% of the general population, with an estimated 1.62 billion people affected[1, 2]. Anemia is a crucial indicator of cancer risk[3], and iron deficiency anemia (IDA) is the most significant contributor, accounting for 50% of all causes of anemia[2].

Numerous epidemiological and experimental studies have shown that iron plays a significant role in cancer development; however, whether iron deficiency (ID) or overload leads to carcinogenesis remains controversial[4–7]. Studies from Japan, the United States, and Finland have associated ID with an increased risk of developing esophageal[6, 7], gastric[8–10], and colon cancers[11–17]. Animal models support that ID accelerates the early development of oral and liver tumors[18, 19] and increases the incidence of colonic and duodenal tumors[20]. By detecting altered iron metabolism in cancer cells, a study concluded that iron contributes to all aspects of tumor growth including tumor initiation, microenvironment, and metastasis[21].

Nevertheless, all relevant studies have focused on the causality between ID and cancer incidence, without accounting for anemia. Therefore, no studies have focused on whether IDA increases the incidence of cancer. Additionally, most related studies have focused on the gastrointestinal tract[6–11, 13–17], except for a study on a Finnish population; however, this particular study only showed that individuals with a high total iron-binding capacity (TIBC) were likely to develop rectal cancer[12]. Therefore, we explored the role of IDA in increasing the cancer risk in other organ systems by classifying all cancers into 22 categories. Moreover, because the aforementioned studies were case—control or nested case—control studies[6–17], we conducted a population-based cohort study to establish a casual relationship between IDA and the increased risk of cancer. We conducted this population-based retrospective cohort study by using a database from the National Health Insurance (NHI) system in Taiwan.

## Materials and Methods

### Data Sources

The NHI program, started in 1995 as a compulsory universal health insurance program, offers comprehensive medical care coverage to all residents of Taiwan, with a coverage rate of up to 98%[22]. The program provides extensive coverage for outpatient, inpatient, emergency, and dental care, as well as prescription drugs. This study used the NHI research database, which is managed and made available to the public by the Taiwan National Health Research Institute (NHRI). The NHI database of catastrophic illness specifically provides comprehensive enrollment information for all patients with severe diseases, such as cancer, who have received copayment exemption under the NHI program. Confidentiality is maintained according to the data regulations of the Bureau of National Health Insurance and the NHRI.

The Taiwan Cancer Registry, which provides data on the incidence rate of cancer, was founded in 1979 by the Department of Health of the executive branch of the central government. The registry is currently operated by the National Public Health Association, and it records newly diagnosed malignant neoplasms from hospitals that provide outpatient or inpatient cancer care.

### Standard Protocol Approvals, Registration, and Patient Consent

The Institutional Review Board of Taipei Veterans General Hospital approved this study (2012-12-013BC). Written patient consent was not required because the NHI data set consists of deidentified secondary data used for research purposes, and the Institutional Review Board issued a formal written waiver regarding the need for consent.

### Study Population

We conducted a retrospective cohort study on the period from January 1, 1996 to December 31, 2011. Patients newly diagnosed with IDA were selected from the period between January 1, 2000 and December 31, 2010. We recruited patients aged 20 years or older at the time of IDA diagnosis who had no prior malignancies. IDA was defined according to the International Classification of Diseases, Ninth Revision, Clinical Modification (ICD-9-CM) code 280.X.

### Statistical Analyses

The main dependent variable was the occurrence of cancer, as reported in the Registry for Catastrophic Illness. For an occurrence of cancer to be reported in the registry, histological confirmation is required for patients diagnosed with cancer. Patients with IDA were followed until the development of cancer, dropout from the NHI program, death, or the end of 2010.

The risk of cancer in the IDA cohort was determined using standardized incidence ratios (SIRs), defined as the observed number of cancer occurrences divided by the expected number of cancer occurrences. The expected number of cancer occurrences was calculated by multiplying the national incidence rate of cancer, stratified by sex, calendar year, and age in 5-year intervals, according to the corresponding stratum-specific person-time accrued in the cohort. The incidence rates of cancer in the general population were obtained from the Taiwan Cancer Registry. The 95% confidence intervals (CIs) for the SIRs were estimated by assuming that the number of cancer occurrences followed a Poisson probability distribution. We determined the SIRs for the subgroups of sex and age. To avoid potential detection bias and the possibility that IDA is a paraneoplastic phenomenon, the subgroups were stratified by the duration when IDA was diagnosed. SIRs were also estimated for each cancer type. To exclude IDA caused by prominent inflammatory diseases that could also increase cancer risk, we conducted a supplemental survey to determine the SIRs of IDA patients by excluding gastroesophageal reflux disease; cholangitis; ulcerative colitis; Crohn’s disease; gastric ulcer; duodenal ulcer; peptic ulcer; gastrojejunal ulcer; chronic hepatitis; Wilson’s disease; alcoholic liver disease; chronic pancreatitis; chronic renal disease; rheumatoid arthritis; diffuse diseases of connective tissue, including systemic lupus erythematosus; tuberculosis; human immunodeficiency virus/acquired immunodeficiency syndrome; syphilis; osteomyelitis; and rheumatic fever.

Data extraction and computation were performed using the Perl programming language (Version 5.12.2). Microsoft SQL Server 2012 (Microsoft Corp., Redmond, WA, USA) was used for data linkage, processing, and sampling. All statistical analyses were performed using SPSS statistical software Version 17.0 for Windows (SPSS, Inc., Chicago, IL, USA). Statistical significance was set at *p* < 0.05.

## Results

### Patient Demographics

A total of 32,390 patients with IDA were identified; 24,610 (75.98%) of whom were female and 7,780 (24.0%) were male. Overall, the cohort was observed for 185,070 person-years from 2000 to 2011. The median follow-up period was 5.43 years [interquartile range (IQR): 2.59–8.80]. The median age of the female patients at diagnosis was 44.15 years (IQR: 34.3–54.05); most of them were diagnosed before 60 years of age (n = 19, 468, 79.1%; Table 1). By comparison, the median age of the male patients at diagnosis was 61.72 years (IQR: 44.75–74.78); 52.59% of them were diagnosed after 60 years of age. Table 1 lists the patient demographics.

**Table 1:** Patient Characteristics.

	Total	Male	Female
No. of patients	32,390	7,780	24,610
Person-years at risk	185,070	36,861	148,208
Median follow-up, years	5.43	3.98	5.89
(interquartile range)	(2.59–8.80)	(1.72–7.62)	(2.96–9.10)
Median age, years	46.27	61.72	44.15
(interquartile range)	(35.94–64.03)	(44.75–74.78)	(34.30–54.05)
Age at diagnosis, years			
20–39	10,693	1,480 (19.02)	9,213 (37.43)
40–59	12,463	2,208 (28.38)	10,255 (41.67)
60–79	6,866	3,063 (39.37)	3,803 (15.45)
≥ 80	2,368	1,029 (13.22)	1,339 (5.44)

### All Cancers

A total of 2,051 patients were diagnosed with cancer within the observation period. Compared with the general population, patients with IDA had an increased overall cancer risk, with an SIR of 2.15 (95% CI: 2.06–2.25; *p* < 0.001). Subgroup analysis for sex showed that both males (SIR: 2.43; 95% CI: 2.27–2.60; *p* < 0.001) and females (SIR: 1.99; 95% CI: 1.88–2.11; *p* < 0.001) had increased SIRs. Similarly, subgroup analysis for age showed that patients in all age groups had increased SIRs (*p* < 0.001). Within the first year of IDA diagnosis, 916 patients were diagnosed with cancer, and the SIR was 5.84 (95% CI: 5.47–6.23; *p* < 0.001). When data from the first year were excluded, the SIR remained significantly elevated at 1.43 (95% CI: 1.34–1.51; *p* < 0.001); when data from the first 5 years were excluded, the SIR remained significantly elevated at 1.30 (95% CI: 1.18–1.43; *p* < 0.001). Table 2 lists the subgroup analysis results. Furthermore, the SIR remained elevated at 1.78 (95% CI: 1.64–1.93; *p* < 0.001) in patients with IDA after exclusion of prominent inflammatory diseases. When data from the first year were excluded, the SIR remained elevated at 1.15 (95% CI: 1.03–1.28; *p* < 0.001). S2 Table presents the results.

**Table 2:** Standardized incidence ratios (SIRs) according to sex, age at diagnosis, and duration of follow-up.

Characteristics	Total			Male			Female		
	Observed	Expected	SIR (95% CI)	Observed	Expected	SIR (95% CI)	Observed	Expected	SIR (95% CI)
All cancers	2,051	952.87	2.15 (2.06–2.25)	846	347.50	2.43 (2.27–2.60)	1205	605.37	1.99(1.88–2.11)
Age									
20–39	130	51.67	2.52 (2.10–2.99)	27	5.47	4.94 (3.25–7.18)	103	46.20	2.23(1.82–2.70)
40–59	705	337.03	2.09 (1.94–2.25)	184	49.60	3.71 (3.19–4.29)	521	287.42	1.81(1.66–1.98)
60–79	877	386.15	2.27 (2.12–2.43)	456	199.26	2.29 (2.08–2.51)	421	186.90	2.25(2.04–2.48)
≥ 80	339	178.01	1.90 (1.71–2.12)	179	93.17	1.92 (1.65–2.22)	160	84.84	1.89(1.60–2.20)
Duration of follow-up (years)									
0–1	916	156.88	5.84 (5.47–6.23)	410	66.58	6.12 (5.57–6.78)	506	90.28	5.60 (5.13–6.11)
≥ 1	1,135	795.98	1.43 (1.34–1.51)	436	280.90	1.55 (1.41–1.70)	699	515.08	1.36 (1.26–1.46)
1–5	707	467.44	1.51 (1.40–1.63)	299	177.51	1.68 (1.50–1.87)	408	289.93	1.41 (1.27–1.55)
≥ 5	428	328.54	1.30 (1.18–1.43)	137	103.39	1.33 (1.11–1.57)	291	225.15	1.29 (1.15–1.50)
Follow-up of duration from disease ≥ 1 year									
All cancers	1,135	795.98	1.43 (1.34–1.51)	436	280.90	1.55 (1.41–1.70)	699	515.08	1.36 (1.26–1.46)
Age									
20–39	68	42.97	1.58 (1.23–2.01)	9	4.56	1.97 (0.90–3.74)	59	38.41	1.54 (1.17–1.98)
40–59	423	292.37	1.45 (1.31–1.59)	86	40.96	2.10 (1.68–2.59)	337	251.41	1.34 (1.20–1.49)
60–79	456	314.55	1.45 (1.32–1.59)	243	159.36	1.52 (1.34–1.73)	213	155.19	1.37 (1.19–1.57)
≥ 80	188	146.10	1.29 (1.11–1.48)	98	76.02	1.29 (1.05–1.57)	90	70.08	1.28 (1.03–1.58)

SIR, standardized incidence ratio; CI, confidence interval

### Specific Cancer Types

To eliminate the influence of a potential detection bias and undetected cancer, SIR analyses were performed after exclusion of patients diagnosed with cancer within 1 year after IDA diagnosis. Patients with IDA, rather than the general population, had a significantly higher risk of developing a subsequent cancer of the following types: leukemia (SIR: 2.51; 95% CI: 1.67–3.63), liver (SIR: 2.27; 95% CI: 1.95–2.62), kidney (SIR: 1.89; 95% CI: 1.33–2.67), esophageal (SIR: 1.80; 95% CI: 1.07–2.85), pancreatic (SIR: 1.75; 95% CI: 1.12–2.61), uterine (SIR: 1.71; 95% CI: 1.19–2.38), bladder (SIR: 1.66; 95% CI: 1.18–2.61), thyroid (SIR: 1.49; 95% CI: 1.05–2.05), colon and rectal (SIR: 1.48; 95% CI: 1.27–1.72), stomach (SIR: 1.43; 95% CI: 1.07–1.87), and head and neck cancer (SIR: 1.33; 95% CI: 1.01–1.72). Table 3 presents the results. After we excluded patients diagnosed with cancer within 5 years after IDA diagnosis, a significantly higher risk of developing a subsequent cancer remained for the following: pancreatic (SIR: 2.31; 95% CI: 1.23–3.95), kidney (SIR: 2.23; 95% CI: 1.32–3.52), liver (SIR: 1.94; 95% CI: 1.48–2.48) and bladder cancers (SIR: 1.74; 95% CI: 1.00–2.83). Table 4 presents the results.

**Table 3:** SIRs of specific cancer types among patients with iron deficiency anemia (IDA) after excluding the first year of observation post IDA.

Site of cancers	Total			Male			Female		
	Observed	Expected	SIR (95% CI)	Observed	Expected	SIR (95% CI)	Observed	Expected	SIR (95% CI)
All cancers	1,135	795.98	1.43 (1.34–1.51)	436	280.90	1.55 (1.41–1.70)	699	515.08	1.36 (1.26–1.46)
Head and neck	58	43.71	1.33 (1.01–1.72)	38	28.17	1.35 (0.95–1.85)	20	15.54	1.29 (0.79–1.99)
Digestive system	467	268.03	1.74 (1.59–1.91)	232	121.06	1.92 (1.68–2.18)	235	146.97	1.60 (1.40–1.82)
Esophagus	18	10.00	1.80 (1.07–2.85)	16	8.06	1.99 (1.14–3.22)	2	1.94	1.03 (0.12–3.73)
Stomach	54	37.74	1.43 (1.07–1.87)	26	17.86	1.46 (0.95–2.13)	28	19.88	1.41 (0.94–2.04)
Colon and rectum	171	115.32	1.48 (1.27–1.72)	75	44.70	1.68 (1.32–2.10)	96	70.62	1.36 (1.10–1.66)
Liver	185	81.50	2.27 (1.95–2.62)	100	41.64	2.40 (1.95–2.92)	85	39.86	2.13 (1.70–2.64)
Extrahepatic biliary tract	15	9.79	1.53 (0.86–2.53)	6	3.40	1.77 (0.65–3.84)	9	6.39	1.41 (0.64–2.67)
Pancreas	24	13.68	1.75 (1.12–2.61)	9	5.40	1.67 (0.76–3.16)	15	8.27	1.81 (1.01–2.99)
Lung and mediastinum	100	93.57	1.07 (0.87–1.30)	55	45.69	1.20 (0.91–1.57)	45	47.89	0.94 (0.69–1.26)
Bone and soft tissue	5	6.16	0.81 (0.26–1.90)	3	2.17	1.38 (0.29–4.04)	2	3.99	0.50 (0.06–1.81)
Skin	24	16.69	1.44 (0.92–2.14)	8	5.97	1.34 (0.58–2.64)	16	10.72	1.49 (0.85–2.42)
Breast	154	137.09	1.12 (0.95–1.32)	0	0.31	0.00 (0.00–11.73)	154	136.77	1.13 (0.96–1.32)
Genitourinary system	192	144.85	1.33 (1.14–1.53)	56	53.91	1.04 (0.78–1.35)	136	90.94	1.50 (1.25–1.77)
Cervix	33	30.99	1.06 (0.73–1.50)	–	–	–	33	30.99	1.06 (0.73–1.50)
Uterus	35	20.43	1.71 (1.19–2.38)	–	–	–	35	20.43	1.71 (1.19–2.38)
Ovaries	18	16.74	1.08 (0.64–1.70)	–	–	–	18	16.74	1.08 (0.64–1.70)
Prostate	30	33.72	0.89 (0.60–1.27)	30	33.72	0.89 (0.60–1.27)	–	–	–
Bladder	39	23.44	1.66 (1.18–2.27)	14	13.38	1.05 (0.57–1.76)	25	10.06	2.48 (1.61–3.67)
Kidney	37	19.54	1.89 (1.33–2.61)	12	6.82	1.76 (0.91–3.07)	25	12.72	1.97 (1.27–2.90)
Thyroid	37	24.87	1.49 (1.05–2.05)	4	1.58	2.53 (0.69–6.49)	33	23.29	1.42 (0.98–1.99)
Hematologic malignancies	63	33.36	1.89 (1.45–2.42)	32	12.57	2.55 (1.74–3.59)	31	20.79	1.49 (1.01–2.12)
Non-Hodgkin's lymphoma	26	17.09	1.52 (0.99–2.23)	9	6.20	1.45 (0.66–2.75)	17	10.88	1.56 (0.91–2.50)
Hodgkin's disease	1	0.75	1.33 (0.03–7.41)	1	0.24	4.23 (0.11–23.57)	0	0.52	0.00 (0.00–7.16)
Multiple myeloma	8	4.37	1.83 (0.79–3.61)	8	1.83	4.36 (1.88–8.59)	0	2.54	0.00 (0.00–1.45)
Leukemia	28	11.15	2.51 (1.67–3.63)	14	4.29	3.26 (1.78–5.47)	14	6.85	2.04 (1.12–3.43)
All others	35	27.65	1.27 (0.88–1.76)	8	9.46	0.85 (0.37–1.67)	27	18.19	1.48 (0.98–2.16)

SIR, standardized incidence ratio; CI, confidence interval

**Table 4:** SIRs of specific cancer types among patients with iron deficiency anemia (IDA) after excluding the first five years of observation post IDA.

Site of cancers	Total			Male			Female		
	Observed	Expected	SIR (95% CI)	Observed	Expected	SIR (95% CI)	Observed	Expected	SIR (95% CI)
All cancers	428	328.54	1.30(1.18–1.43)	137	103.39	1.33(1.11–1.57)	291	225.15	1.29(1.15–1.45)
Head and neck	20	17.29	1.16(0.71–1.79)	9	10.65	0.85(0.39–1.60)	11	6.65	1.65(0.83–2.96)
Digestive system	158	107.25	1.47(1.25–1.72)	65	44.09	1.47(1.14–1.88)	93	63.16	1.47(1.19–1.80)
Esophagus	5	3.82	1.31(0.43–3.06)	4	2.98	1.34(0.37–3.44)	1	0.84	1.19(0.03–6.64)
Stomach	20	14.33	1.40(0.85–2.16)	8	6.19	1.29(0.56–2.55)	12	8.14	1.47(0.76–2.57)
Colon and rectum	54	47.54	1.14(0.85–1.48)	23	16.66	1.38(0.87–2.07)	31	30.88	1.00(0.68–1.43)
Liver	62	32.04	1.94(1.48–2.48)	27	15.01	1.80(1.19–2.62)	35	17.02	2.06(1.43–2.86)
Extrahepatic biliary tract	4	3.90	1.03(0.28–2.63)	0	1.22	0.00(0.00–3.01)	4	2.67	1.50(0.41–3.83)
Pancreas	13	5.63	2.31(1.23–3.95)	3	2.02	1.49(0.31–4.34)	10	3.61	2.77(1.33–5.10)
Lung and mediastinum	46	37.99	1.21(0.89–1.61)	24	16.66	1.44(0.92–2.14)	22	21.34	1.03(0.65–1.56)
Bone and soft tissue	1	2.50	0.40(0.01–2.23)	1	0.81	1.23(0.03–6.85)	0	1.69	0.00(0.00–2.18)
Skin	11	6.84	1.61(0.80–2.88)	6	2.27	2.64(0.97–5.75)	5	4.57	1.09(0.36–2.55)
Breast	70	62.14	1.13(0.88–1.42)	0	0.11	0.00(0.00–32.94)	70	62.03	1.13(0.88–1.43)
Genitourinary system	74	59.05	1.25(0.98–1.57)	23	20.25	1.14(0.72–1.70)	51	38.80	1.31(0.98–1.73)
Cervix	12	11.81	1.02(0.53–1.77)	–	–	–	12	11.81	1.02(0.53–1.77)
Uterus	10	9.84	1.02(0.49–1.87)	–	–	–	10	9.84	1.02(0.49–1.87)
Ovaries	5	7.32	0.68(0.22–1.59)	–	–	–	5	7.32	0.68(0.22–1.59)
Prostate	13	12.83	1.01(0.54–1.73)	13	12.83	1.01(0.54–1.73)	0	–	–
Bladder	16	9.18	1.74(1.00–2.83)	5	4.88	1.02(0.33–2.39)	11	4.30	2.56(1.28–4.58)
Kidney	18	8.07	2.23(1.32–3.52)	5	2.53	1.97(0.64–4.60)	13	5.54	2.35(1.25–4.01)
Thyroid	16	11.17	1.43(0.82–2.33)	0	0.63	0.00(0.00–5.88)	16	10.54	1.52(0.87–2.47)
Hematologic malignancies	18	13.67	1.32(0.78–2.08)	5	4.67	1.07(0.35–2.50)	13	9.00	1.44(0.77–2.47)
Non-Hodgkin's lymphoma	10	7.06	1.42(0.68–2.60)	2	2.31	0.86(0.10–3.12)	8	4.75	1.69(0.73–3.32)
Hodgkin's disease	0	0.29	0.00(0.00–12.73)	0	0.09	0.00(0.00–42.70)	0	0.20	0.00(0.00–18.13)
Multiple myeloma	1	1.82	0.55(0.01–3.07)	1	0.68	1.46(0.04–8.15)	0	1.13	0.00(0.00–3.26)
Leukemia	7	4.50	1.55(0.62–3.20)	2	1.59	1.26(0.15–4.55)	5	2.92	1.71(0.56–4.00)
All others	14	10.63	1.32(0.72–2.21)	4	3.25	1.23(0.33–3.15)	10	7.37	1.36(0.65–2.49)

SIR, standardized incidence ratio; CI, confidence interval

## Discussion

Based on our research, this is the first population-based cohort study to evaluate the risk of all cancer types among patients with IDA. Our study included 32,390 patients with a follow-up of 185,070 person-years. The study data showed that compared with the general population, patients with IDA had an increased overall cancer risk (SIR: 2.15), with SIRs of 2.43 and 1.99 for males and females, respectively. After we excluded patients diagnosed with cancer in the first year and first 5 years, the remaining patients still showed a significantly increased cancer risk with SIRs of 1.43 and 1.30, respectively.

The study design included unbiased subject selection and age-, sex-, and calendar-year-matched SIR estimates. Because participation in the NHI is compulsory and all residents of Taiwan can access healthcare with low copayments, referral bias is essentially removed and follow-up is complete. To apply for a cancer catastrophic illness certificate, pathologic proof of malignancy is mandatory, and relevant reports of laboratory and imaging studies should be provided. Patients with a certificate of catastrophic illness can be exempted from related medical expenses, particularly hospital costs. Therefore, cancer diagnoses in this study were reliable and exhaustive.

In this study, the number of women diagnosed with IDA was three times that of men diagnosed with IDA. Furthermore, the median age of the female patients at IDA diagnosis was 44.15 years; most of them were diagnosed before 60 years of age. By comparison, the median age of the male patients at IDA diagnosis was 61.72 years; 52.59% of whom were diagnosed after 60 years of age. These findings are comparable with those of a study in which IDA was more prevalent in women, particularly in those of childbearing age[23]. In addition, the prevalence rate of anemia rises more rapidly from middle age in men than in women, has a higher prevalence rate in men aged ≥75 years than in women of the same age, and is highest in men aged ≥85 years[24].

We found that the overall cancer risk in IDA patients was higher than that in the general population, with an SIR of 2.15. Although ID is a major cause of anemia, it also impairs the molecular and metabolic functions of cells. By causing mitochondrial dysfunction[25], cells with ID undergo apoptosis inhibition[26], genomic instability and aging[25], and ID-induced reduction in nitric oxide synthase activity[27], resulting in DNA damage, oncogene activation, DNA repair enzyme inhibition[28], and macrophage antitumor activity downregulation[27]. ID also causes lymphocyte DNA damage by generating excess reactive oxygen species through elevated oxidant levels and decreased antioxidant enzyme activities[29], leading to human cell carcinogenesis[30]. These mechanisms might contribute differently to specific stages of cancer development and to certain cancer types. Moreover, several studies have shown that IDA alters immune activities including both cellular and humoral immunity[31–34], thus creating a microenvironment permissive for carcinogenesis. Because a hallmark of cancer cells is the ability to evade immune destruction[35], IDA may subtly change the microenvironment of the human body, which may become more vulnerable to cancer cells.

In the present study, the incidence of cancer increased in the first year after IDA diagnosis and showed an SIR of 5.84. Detection bias may be a possible explanation for this finding[36]. Compared with the general population, patients with IDA, particularly men or nonmenstruating women, are more likely to undergo diagnostic tests or have more outpatient follow-up visits, thus leading to an earlier diagnosis of cancer. In addition, an increased SIR might indicate that anemia is a manifestation of occult cancer[37]. However, the SIRs in the present study remained significantly elevated despite the exclusion of data from the first year (SIR: 1.43) and first 5 years (SIR: 1.30) of cancer diagnosis; therefore, it is reasonable to conclude that the increased cancer risk in patients with IDA was not due only to the aforementioned reasons.

In our study, excluding patients diagnosed with IDA and cancer within the first 5 years revealed increased risks of cancers of the pancreas (SIR: 2.31), kidney (SIR: 2.23), liver (SIR: 1.94), and bladder (SIR: 1.74), which remained significantly high. Based on our research, no previous studies have shown a relationship between IDA and the increased risk of these cancers. The result might be explained by the aforementioned mechanisms, but future studies are required to establish a clearer understanding of the relationship between IDA and the increased risk of cancer.

An increased colorectal and stomach cancer risk could be associated with chronic diseases such as ulcerative colitis or gastritis[38]; therefore, we conducted a supplemental survey after excluding the prominent inflammatory diseases listed in the Statistical Analyses of **Materials and Methods**. The SIR remained elevated at 1.14 after the data from the first year were excluded, thus demonstrating that cancer risk is increased in IDA patients without chronic inflammatory diseases.

Our study has several limitations. First, several cancer-related demographic variables, such as family history of cancer[39], environmental exposure[40], diet[41], cigarette smoking[42], and alcohol use[43] were unavailable. Second, the diagnostic accuracy and severity of IDA could not be determined. Whether the severity of IDA is related to carcinogenesis should be further evaluated. Third, patients with IDA generally receive treatment with iron supplements; however, we did not consider the treatment of IDA while establishing the relationship between IDA and an increased cancer risk. Fourth, our choice of inflammatory diseases being excluded may be questionable because we could not exclude all inflammatory diseases; the relationship between an increased cancer risk and several of the excluded diseases requires further establishment. Finally, the follow-up period of our study might be too short to detect the carcinogenesis of certain types of cancer.

In conclusion, this population-based retrospective cohort study presents an increased cancer risk in patients with IDA. Specifically, the risks of pancreatic, kidney, liver, and bladder cancers significantly increased after we excluded patients diagnosed with IDA and cancer within the first 5 years. The increased cancer risk in patients with IDA may be caused by altered immune activities because of IDA. Additional large, population-based prospective studies are required to investigate the relationship between IDA and cancer risk.

## Supporting Information

S1 Table Characteristics of patients after exclusion of inflammatory disease. (DOC)

S2 Table Standardized incidence ratios (SIRs) according to sex, age at diagnosis and duration of follow-up after excluding inflammatory disease. (DOC)
