# Correction: Risk of Cancer in Patients with Iron Deficiency Anemia: A Nationwide Population-Based Study

**DOI:** 10.1371/journal.pone.0125951

**Published:** 2015-04-23

**Authors:** The PLOS ONE Staff

The authors are listed out of order, and the first three authors, Ning Hung, Li-Yu Hu, and Cheng-Che Shen, should be noted as contributing equally to this work. Please view the correct author order, affiliations, and citation here:

Ning Hung^2¶^, Li-Yu Hu^1¶^, Cheng-Che Shen^3,4^, Yu-Wen Hu^2,5,6^, Chiu-Mei Yeh^7^, Chung-Jen Teng^2,6,8^, Ai-Seon Kuan^2^, San-Chi Chen^9^, Tzeng-Ji Chen^2,7^, Chia-Jen Liu^2,6,9^*

1 Department of Psychiatry, Kaohsiung Veterans General Hospital, Kaohsiung, Taiwan, 2 School of Medicine, National Yang-Ming University, Taipei, Taiwan, 3 Department of Psychiatry, Chiayi Branch, Taichung Veterans General Hospital, Chiayi, Taiwan, 4 Department of information management, National Chung-Cheng University, Chiayi, Taiwan, 5 Cancer Center, Taipei Veterans General Hospital, Taipei, Taiwan, 6 Institute of Public Health, National Yang-Ming University, Taipei, Taiwan, 7 Department of Family Medicine, Taipei Veterans General Hospital, Taipei, Taiwan, 8 Division of Oncology and Hematology, Department of Medicine, Far Eastern Memorial Hospital, New Taipei City, Taiwan, 9 Division of Hematology and Oncology, Department of Medicine, Taipei Veterans General Hospital, Taipei, Taiwan

¶These authors contributed equally to this work.

*Corresponding author

E-mail: pures1000@yahoo.com.tw
