# Supplementary material for: Risk of Cancer in Patients with Iron Deficiency Anemia: A Nationwide Population-Based Study
Source: PLoS One. 2015 Mar 17;10(3):e0119647. doi: 10.1371/journal.pone.0119647 (PMC4363660; doi:10.1371/journal.pone.0119647)
Supplement: S1 Table — (DOC) [file pone.0119647.s001.doc]

**S1 Table. Characteristics of patients after exclusion of inflammatory disease**

|  | | Total | Mal Male | Female |
| --- | --- | --- | --- | --- |
| No. of patients | | 15048 | 2739 | 12309 |
| Person–years at risk | | 101756 | 17364 | 84391.7 |
| Median follow–up, years | | 7.19 | 6.76 | 7.29 |
|  | (interquartile range) | (3.79–9.80) | (3.06–9.60) | (3.97–9.85) |
| Median age, years | | 38 | 41 | 38 |
|  | (interquartile range) | (26–48) | (22–63) | (26–47) |
| Age at diagnosis, years | |  |  |  |
|  | 0–19 | 2212 | 618 | 1594 |
|  | 20– 39 | 5852 | 728 | 5124 |
|  | 40–59 | 5083 | 614 | 4469 |
|  | 60–79 | 1418 | 600 | 818 |
|  | ≥ 80 | 483 | 179 | 304 |
