# Supplementary material for: Risk of Cancer in Patients with Iron Deficiency Anemia: A Nationwide Population-Based Study
Source: PLoS One. 2015 Mar 17;10(3):e0119647. doi: 10.1371/journal.pone.0119647 (PMC4363660; doi:10.1371/journal.pone.0119647)
Supplement: S2 Table — (DOC) [file pone.0119647.s002.doc]

**S2 Table. Standardized incidence ratios (SIRs) according to sex, age at diagnosis and duration of follow-up after excluding inflammatory disease.**

|  | Total |  |  | Male |  |  | Female |  | |  |
| --- | --- | --- | --- | --- | --- | --- | --- | --- | --- | --- |
| Characteristics | Observed | Expected | SIR (95% CI) | Observed | Expected | SIR (95% CI) | Observed | Expected | | SIR (95% CI) |
| All cancers | 592 | 332.34 | 1.78(1.64–1.93) | 190 | 85.15 | 2.23(1.93–2.57) | 402 | 247.19 | | 1.63(1.47–1.79) |
| Age |  |  |  |  |  |  |  |  | |  |
| 0 – 19 | 5 | 1.32 | 3.78(1.23–8.82) | 3 | 0.52 | 5.72(1.18–16.71) | 2 | 0.80 | | 2.51(0.30–9.06) |
| 20 – 39 | 70 | 32.14 | 2.18(1.70–2.75) | 7 | 3.22 | 2.18(0.87–4.48) | 63 | 28.92 | | 2.18(1.67–2.79) |
| 40 – 59 | 261 | 159.20 | 1.64(1.45–1.85) | 47 | 16.70 | 2.81(2.07–3.74) | 214 | 142.50 | | 1.50(1.31–1.72) |
| 60 – 79 | 172 | 98.85 | 1.74(1.49–2.02) | 94 | 46.24 | 2.03(1.64–2.49) | 78 | 52.61 | | 1.48(1.17–1.85) |
| ≥ 80 | 84 | 40.83 | 2.06(1.64–2.55) | 39 | 18.47 | 2.11(1.50–2.89) | 45 | 22.36 | | 2.01(1.47–2.69) |
| Duration of follow-up (years) | | |  |  |  |  |  |  | |  |
| 0 – 1 | 259 | 42.43 | 6.10(5.38–6.89) | 101 | 12.97 | 7.79(6.34–9.46) | 158 | 29.47 | | 5.36(4.56–6.27) |
| ≥ 1 | 333 | 289.90 | 1.15(1.03–1.28) | 89 | 72.19 | 1.23(0.99–1.52) | 244 | 217.72 | | 1.12(0.98–1.27) |
| 1 – 5 | 189 | 148.99 | 1.27(1.09–1.46) | 54 | 39.97 | 1.35(1.01–1.76) | 135 | 109.02 | | 1.24(1.04–1.47) |
| ≥ 5 | 144 | 140.92 | 1.02(0.86–1.20) | 35 | 32.02 | 1.09(0.76–1.52) | 109 | 108.69 | | 1.00(0.82–1.21) |
| Follow-up of duration from disease ≥ 1 year | | | |  |  |  |  | |  |  |
| All cancers | 333 | 289.90 | 1.15(1.03–1.28) | 89 | 72.19 | 1.23(0.99–1.52) | 244 | | 217.72 | 1.12(0.98–1.27) |
| Age |  |  |  |  |  |  |  | |  |  |
| 0 – 19 | 2 | 1.03 | 1.94(0.23–7.01) | 1 | 0.44 | 2.27(0.06–12.67) | 1 | | 0.59 | 1.69(0.04–9.42) |
| 20 – 39 | 42 | 27.54 | 1.53(1.10–2.06) | 4 | 2.81 | 1.43(0.39–3.65) | 38 | | 24.73 | 1.54(1.09–2.11) |
| 40 – 59 | 163 | 141.71 | 1.15(0.98–1.34) | 17 | 14.42 | 1.18(0.69–1.89) | 146 | | 127.29 | 1.15(0.97–1.35) |
| 60 – 79 | 84 | 84.61 | 0.99(0.79–1.23) | 50 | 38.75 | 1.29(0.96–1.70) | 34 | | 45.86 | 0.74(0.51–1.04) |
| ≥ 80 | 42 | 35.01 | 1.20(0.86–1.62) | 17 | 15.77 | 1.08(0.63–1.73) | 25 | | 19.25 | 1.30(0.84–1.92) |

SIR Standardized incidence ratio; CI confidence interval
